# Utility of Colonoscopy After Acute Colonic Diverticulitis

**DOI:** 10.3390/diagnostics16071051

**Published:** 2026-04-01

**Authors:** Miguel Suárez, Raquel Martínez, Ana M. Torres, Jorge Mateo

**Affiliations:** 1 Gastroenterology Department, University Hospital of Cuenca, 16004 Cuenca, Spain; 2 Medical Analysis Expert Group, Instituto de Investigación Sanitaria de Castilla-La Mancha (IDISCAM), 45071 Toledo, Spain; 3 Medical Analysis Expert Group, Institute of Technology, Universidad de Castilla-La Mancha, 16071 Cuenca, Spain

**Keywords:** colonic diverticulitis, colonic diverticulosis, colonoscopy, colorectal cancer, screening

## Abstract

Acute diverticulitis (AD) is a common gastrointestinal condition that has seen a significant rise in incidence and prevalence, largely due to the aging population and the increasing prevalence of obesity. Historically, routine colonoscopy was recommended after every episode of AD, but recent studies and meta-analyses have questioned this practice. Evidence now supports a more selective approach, suggesting that colonoscopy should only be performed in patients with complicated AD, persistent alarm symptoms (abdominal pain, weight loss, altered bowel habits, blood in stool, or iron-deficiency anemia), or imaging findings suggestive of neoplasia. For uncomplicated AD without alarm symptoms, routine colonoscopy is not justified, as it may lead to unnecessary complications and overuse of healthcare resources. Current guidelines reflect this shift, recommending individualized decision-making based on the patient’s clinical history and risk factors. Emerging non-invasive diagnostic tools, such as fecal immunochemical tests (FIT) and artificial intelligence (AI)-based models, hold promise for improving risk stratification and potentially reducing the need for invasive procedures. This narrative review, based on a structured literature search, synthesizes the evolution of post-AD colonoscopy recommendations, presents current evidence, and highlights future research directions on alternative diagnostic methods and their potential to optimize patient care and clinical decision-making.

## 1. Introduction

Colonic diverticulosis is a common and increasing condition, particularly due to the rising life expectancy [1]. Although commonly referred to as diverticula, these are technically pseudodiverticula, as they form when the mucosa and submucosa of the colon protrude through weak points in the muscularis propria. These weak points are often associated with areas where blood vessels penetrate and supply mucosa and submucosa. The main distinction from true diverticula is that the latter contain all layers of the intestinal wall, whereas pseudodiverticula lack a complete muscular layer [2].

In Western countries, it is estimated that more than 50% of the population over the age of 60 has diverticulosis [3,4]. Although traditionally associated with Western nations, the prevalence of diverticular disease has been increasing in recent decades. The Westernization of Asian countries in terms of diet, along with immigration, has led to a rise in its prevalence and complications in these regions [5]. In Japan, for example, its prevalence increased from 13% in the 1990s to nearly 24% in the following decade [6]. In Africa, the prevalence has also risen, from 3.5% to 9.7% over the past 40 years [7].

Numerous risk factors for the development of diverticula have been described, with age being the most well-known and strongly associated, especially after the age of 60, as mentioned earlier [8]. Other factors include male sex, obesity, alcohol and tobacco consumption, low-fiber diet, hypertension, and the use of certain medications such as corticosteroids and non-steroidal anti-inflammatory drugs (NSAIDs) [9,10]. Genetic factors also play a significant role. Genome-wide association studies (GWAS) investigating diverticulosis have identified nearly 50 risk loci associated with intestinal neuromuscular function, connective fiber support, epithelial dysfunction, and tissue remodeling as a primary mechanism for diverticulum formation, with genes such as *CCN3, CRISPLD2, ENTPD7, PHGR1*, and *TNFSF13* showing potential causal effects on diverticulosis [11,12].

The most common complication of diverticulosis is acute diverticulitis (AD), which involves inflammation of the diverticula and can lead to potentially severe complications. Traditionally, the development of AD has been associated with the presence of advanced colonic lesions and colorectal cancer (CRC). As a result, colonoscopy is often requested following an episode of acute diverticulitis. In this review, we will discuss the utility of colonoscopy for detecting colonic lesions after an episode of AD. We will first review the terminology within the spectrum of diverticular disease to clarify the terms used throughout the paper. Special emphasis will be placed on the subclassification of diverticulitis, briefly discussing the diagnosis and treatment of each condition. Finally, a literature review of the most recent studies will be conducted to assess in which cases colonoscopy should be indicated.

A narrative, non-systematic literature review was conducted to identify relevant evidence regarding the role of colonoscopy after an episode of acute colonic diverticulitis. Electronic searches were performed in PubMed/MEDLINE and Embase for articles published between January 2000 and December 2024.

The search strategy included combinations of the following keywords and Medical Subject Headings (MeSH) terms: “acute diverticulitis”, “colonoscopy”, “colorectal cancer”, “follow-up”, and “computed tomography”.

Priority was given to systematic reviews, meta-analyses, clinical practice guidelines, and large observational studies published in English. Additional relevant publications were identified through manual screening of the reference lists of key articles.

The selected literature was analyzed qualitatively to summarize current evidence and evolving recommendations regarding post-diverticulitis colonoscopy.

## 2. Spectrum of Diverticular Disease and Pathophysiology

Diverticular disease encompasses a broad spectrum of conditions ranging from diverticulosis to the complications resulting from diverticulitis. The following are the different stages described in the literature:

- **Diverticulosis**

This term refers to the presence of colonic diverticula without implying disease. Most individuals with diverticulosis remain asymptomatic throughout their lives [13,14].

- **Symptomatic uncomplicated diverticular disease (SUDD)**

SUDD is a controversial entity without a universally accepted definition [14]. It describes the presence of symptoms such as abdominal pain, bloating, and altered bowel habits in patients with diverticulosis in the absence of overt inflammation or bleeding. Because these symptoms overlap with those of irritable bowel syndrome (IBS), differentiation between both conditions may be challenging. In addition, the lack of standardized diagnostic criteria has led to widely variable prevalence estimates, ranging from approximately 25% in earlier reports to 6.81% in more recent analyses from the same research group [15,16].

- **Peridiverticular Colitis or Segmental Colitis Associated with Diverticulosis (SCAD)**

This condition is characterized by chronic inflammation of the colonic mucosa in segments affected by diverticulosis, most commonly in the sigmoid colon. Although it may resemble inflammatory bowel disease, particularly ulcerative colitis, it can usually be distinguished by the clinical context, segmental involvement, and the absence of deep ulcerations. Histologically, findings may include inflammatory infiltrates in the lamina propria, neutrophilic cryptitis, crypt abscesses, basal lymphoid aggregates, architectural distortion, and Paneth cell metaplasia [17].

Clinically, patients may present with abdominal pain, diarrhea, hematochezia, and tenesmus, while fever is uncommon and may help differentiate this condition from diverticulitis [18]. Peridiverticular colitis and diverticulitis may coexist, and SCAD has been suggested as a potential precursor of acute diverticulitis, although the risk of progression remains unclear [19].

- **Acute diverticulitis**

AD is commonly described as inflammation or infection of diverticula; however, the term more accurately refers to inflammation involving the peridiverticular tissues and the surrounding colonic wall [20]. It represents the most common clinical manifestation of colonic diverticular disease and constitutes a significant healthcare burden in terms of hospital admissions, complications, and the need for surgical intervention. Its incidence has increased in recent decades, particularly in Western countries and among younger individuals compared with traditional epidemiological patterns. Despite the high prevalence of diverticulosis, the risk of developing AD is estimated to be slightly above 4% among individuals with diverticula [3].

Traditionally, the pathogenesis of AD has been attributed to obstruction of the diverticular neck by fecaliths or food residues, leading to increased intraluminal pressure, bacterial proliferation, and localized inflammation, which may ultimately result in microperforation of the diverticulum and inflammation of the surrounding colonic wall [21]. However, current evidence indicates that this process is likely multifactorial, involving interactions between environmental exposures, host susceptibility, and alterations in the intestinal microbiome.

- **Genetic susceptibility**

A growing body of evidence supports a significant genetic component in diverticular disease, with heritability estimates of approximately 40–50% [19]. Twin and sibling studies have demonstrated that the risk of diverticular disease is roughly threefold higher among siblings of affected individuals, with a stronger concordance observed in monozygotic twins compared with dizygotic twins [22].

Large GWAS have identified numerous susceptibility loci associated with diverticular disease, many of which involve genes related to extracellular matrix organization, smooth muscle contractility, and immune regulation. More than 30 loci associated with diverticular disease have been described, with variants in genes such as *PHGR1, FAM155A, CALCB*, and *S100A10* showing strong associations with the risk of developing diverticulitis [23,24]. Additional studies have also highlighted variants in genes related to connective tissue structure and RhoGTPase signaling pathways, including *ARHGAP15* and *COL3A1*, further supporting the hypothesis that structural vulnerability of the colonic wall contributes to diverticulum formation and subsequent inflammation [25,26].

Recent multi-ancestral meta-analyses have expanded the genetic landscape of diverticular disease to more than one hundred susceptibility loci, with common variants explaining approximately 30–35% of the estimated heritability. These studies consistently prioritize cell types such as fibroblasts, smooth muscle cells, and interstitial cells of Cajal, reinforcing the concept that alterations in colonic wall structure and motility predispose individuals to diverticular disease and its complications [27].

The integration of these genetic findings into polygenic risk scores has enabled the identification of individuals with increased susceptibility to diverticulitis. Several studies have demonstrated that higher polygenic risk scores are associated with a substantially increased likelihood of developing AD and, in some cases, with a greater lifetime disease burden. Although these scores retain predictive value across different ancestral populations, their performance may vary, highlighting the importance of considering population-specific genetic backgrounds [27,28].

- **Role of the intestinal microbiome**

Increasing evidence also suggests that the intestinal microbiome plays a relevant role in the transition from diverticulosis to acute diverticulitis, although findings remain heterogeneous. Several studies have reported reduced microbial diversity in patients with diverticulitis, together with a shift from commensal short-chain fatty acid-producing bacteria, such as members of *Lachnospiraceae*, *Ruminococcus*, and *Faecalibacterium*, toward increased abundance of potentially pro-inflammatory taxa, including *Fusobacteria*, *Prevotella*, *Paraprevotella*, and certain *Enterobacteriaceae* [29].

A recent case–control study in women demonstrated differences in microbiome composition and metabolic activity between patients with diverticulitis and healthy controls. In this study, individuals with AD exhibited increased levels of pro-inflammatory bacteria such as *Ruminococcus gnavus* and reduced abundance of butyrate-producing bacteria, supporting a potential role of microbial dysbiosis in the inflammatory process [30].

Additional studies have reported reductions in anti-inflammatory *Clostridium* clusters and lower abundance of *Lactobacilli* and *Bacteroides*, together with relative overgrowth of taxa such as *Bifidobacteria*, *Akkermansia*, and *Proteobacteria*, suggesting the presence of a pro-inflammatory microbial profile in symptomatic diverticular disease. These microbial alterations may promote mucosal immune activation, epithelial barrier dysfunction, and local inflammatory responses [31,32].

Recent pathophysiological models propose that fecal stasis within diverticula and delayed colonic transit may further promote changes in microbial metabolism, leading to altered production of metabolites such as secondary bile acids, short-chain fatty acids, sulfur-containing compounds, and ceramides. These metabolites may damage the mucosal barrier and amplify inflammatory signaling within the peridiverticular environment. Integrative multi-omic studies have identified associations between specific microbes, including *Bilophila wadsworthia*, and metabolic pathways related to sulfur metabolism, bile acid profiles, and ceramide production, suggesting that microbial metabolic activity, rather than the presence of individual taxa alone, may play an important role in the development of diverticulitis [33].

Nevertheless, results remain inconsistent, and several population-based studies have not identified clear differences in overall microbiome composition between individuals with asymptomatic diverticulosis and healthy controls. This observation suggests that relevant dysbiosis may be localized to the mucosal or peridiverticular niche or may occur dynamically around the time of the acute inflammatory episode. Targeted modulation of the microbiome through dietary interventions, antibiotics, or probiotics has been proposed as a potential preventive or therapeutic strategy. Some studies suggest that probiotic strains with anti-inflammatory properties may reduce symptom severity or shorten hospitalization in uncomplicated diverticulitis, although high-quality clinical evidence remains limited and heterogeneous [34,35].

- **Environmental and lifestyle risk factors**

In addition to genetic susceptibility and microbiome alterations, several environmental and lifestyle factors have been associated with an increased risk of AD. These include low dietary fiber intake, obesity, smoking, alcohol consumption, sedentary lifestyle, and the use of certain medications, such as NSAIDs [19]. Many of these factors overlap with those associated with the development of diverticulosis, although age remains the most important non-modifiable risk factor.

Importantly, older recommendations advising patients to avoid nuts, corn, and fruits with seeds have been refuted, as current evidence indicates that consumption of these foods does not increase the risk of diverticulitis [36].

## 3. Diagnosis and Clinical Classification of Acute Diverticulitis

The diagnosis of AD can be challenging, as clinical evaluation alone may miss up to 50% of cases [20]. Therefore, diagnosis should combine clinical findings with laboratory parameters and imaging studies.

Clinically, acute left-sided diverticulitis (ALCD) typically presents with lower left quadrant abdominal pain, often accompanied by low-grade fever and changes in bowel habits. However, diverticula may occur throughout the colon, and symptoms can arise in other abdominal quadrants depending on the affected segment. In contrast, painless lower gastrointestinal bleeding is more commonly related to diverticular bleeding rather than diverticulitis and does not predict the development of AD [20]. Diverticular bleeding occurs more frequently in elderly patients and usually originates in the right colon [37].

Among laboratory markers, C-reactive protein (CRP) has been widely studied as an indicator of disease severity. Although no universal cutoff value has been established, higher CRP levels are consistently associated with an increased likelihood of complicated diverticulitis and adverse outcomes [38].

Imaging plays a central role in confirming the diagnosis, particularly during the first episode. Abdominal computed tomography (CT) is the preferred modality due to its sensitivity and specificity above 90% and its ability to identify complications such as abscesses, perforation, or fistulas [39]. In selected patients with suspected mild or recurrent disease, abdominal ultrasound (US) may be considered as an alternative, offering advantages such as the absence of radiation and bedside availability.

From a practical perspective, AD is classified into uncomplicated and complicated forms, which guide management:

- **Uncomplicated AD**

It involves inflammation confined to the diverticulum and the adjacent colonic wall and is usually managed conservatively with bowel rest, hydration, and analgesia. Routine antibiotic therapy is not required in all cases, as randomized studies have not demonstrated clear benefits [19,20].

- **Complicated AD**

It occurs when inflammation extends beyond the colonic wall and is associated with complications such as abscesses, perforation, fistulas, or obstruction [40,41]. These patients may present with similar symptoms but often show signs of peritonitis and more pronounced laboratory abnormalities. Approximately 10–15% of patients develop complicated disease, and classification systems such as the Hinchey classification are commonly used to stratify severity and guide management. Table 1 presents this classification and its management.

In some patients, the inflammatory process does not fully resolve after an acute episode, leading to chronic diverticulitis, characterized by persistent abdominal pain and altered bowel habits resembling irritable bowel syndrome. Ongoing inflammation may cause damage to local sensory and motor nerves and alterations in the intestinal microbiota [42]. Although its epidemiology is not well defined, approximately 20% of patients with AD experience at least one recurrence within 10 years [21]. Chronic diverticulitis is considered uncomplicated when only colonic wall thickening or mucosal inflammation is present, whereas the development of stenosis with obstruction or fistula formation defines complicated chronic disease [20].

## 4. Utility of Colonoscopy After an Episode of Acute Diverticulitis

Within endoscopy services, a frequent indication for colonoscopy is the evaluation of advanced colonic lesions or CRC following an episode of AD. Although we have not found specific data on what percentage of total endoscopies this represents, anyone who regularly performs colonoscopies is aware of this reality. For example, a multicenter retrospective study published by Singh et al., which reviewed AD episodes over 12 years, found that nearly 91% of patients underwent a colonoscopy after this event. Of these, only 1.28% had a diagnosis of CRC, resulting in nearly 5000 colonoscopies during this period [47]. Another study reported that colonoscopy was performed in 48.5% of patients after an episode of AD, a significantly lower percentage but still noteworthy [48].

It is important to note that, increasingly, endoscopy services are under greater workload pressure, and waiting lists are growing [49]. This necessitates improvements in the indications for endoscopy and the appropriate selection of patients for these procedures by all healthcare providers. This ensures that patients requiring an endoscopy for suspected tumoral lesions or other significant pathologies, such as inflammatory bowel disease, can benefit from timely procedures without long waits. Furthermore, although the widespread use of sedation has made colonoscopy an acceptable procedure for the general population, we must not forget that it is an invasive test that carries its own risks and potential complications [50].

Traditionally, there has been a mandatory concept of performing a colonoscopy after an episode of acute diverticulitis, primarily to rule out CRC. Studies from researchers such as Boulos et al. and Irvine et al. in the 1980s supported this approach [51,52]. Additionally, studies on patients with diverticulitis who also presented diverticular bleeding or post-diverticulitis stenosis further reinforced the indication for colonoscopy in these cases [53,54]. Even later, clinical guidelines on the management of diverticular disease in the 2000s relied on this published evidence to recommend routine colonoscopy under these circumstances [55,56,57]. More recently, for example, the European Society of Colorectal Surgery (ESCS) in 2014 supported colonoscopy after an episode of AD [58]; and the Italian Society of Colon and Rectal Surgery in 2015 also emphasized the need for this procedure [59]. Other cohort studies and some meta-analyses, generally retrospective and led by specialists from various disciplines, continued to evaluate this indication, maintaining or suggesting the routine recommendation for colonoscopy based on their findings [60,61,62].

Improvements in imaging techniques such as US and CT, particularly the latter, as well as advancements in the quality of endoscopic imaging, better endoscopist training, and the increasing number of colonoscopies performed for various reasons, including colorectal cancer screening programs, have led to reconsideration of this longstanding approach.

One of the first published works questioning the routine performance of colonoscopy after an episode of AD was presented in 2004 by Anglade et al. at a meeting of the American Society for Gastrointestinal Endoscopy (ASGE). This study, aimed at comparing the presence of advanced lesions between a group of patients who had experienced an episode of AD and a group undergoing CRC screening, found no significant difference in the detection of relevant lesions between the two groups [63]. Over the past decade, evidence supporting this view has steadily increased, seriously challenging the traditional practice of routinely performing colonoscopy following an AD episode.

An elegant article published by Perry and Sandler in 2013, which re-evaluated the conventional wisdom regarding diverticular disease, also began to question this assumption. Although they continued to support the performance of colonoscopy according to the American College of Gastroenterology (ACG) guidelines, they pointed out limitations in the available evidence, such as the exclusion in some studies of patients who had undergone colonoscopy within the previous year, thereby raising doubts about the need for the procedure in such patients [64].

Prior to that publication, a systematic review compared the prevalence of CRC in patients diagnosed with AD by CT scan and subsequently undergoing surgery, colonoscopy, or barium enema within six months of diagnosis versus the general population. The review demonstrated no significant differences in CRC diagnosis between the two groups, concluding that the available data were insufficient to recommend routine colonoscopy following AD diagnosis [65]. Another subsequent systematic review evaluating CRC detection after an episode of uncomplicated diverticulitis concluded that routine colonoscopy in these cases was unnecessary [66]. A systematic review and meta-analysis by Sharma et al., including nearly 2000 patients from seven different countries, also concluded that after an episode of uncomplicated diverticulitis confirmed by imaging, in the absence of other symptoms, colonoscopy might not be required [67]. A more recent meta-analysis published by Rottier et al., focusing exclusively on episodes of left-sided diverticulitis, similarly concluded that if CT imaging showed only uncomplicated diverticulitis without other abnormalities, and in the absence of alarm symptoms, these patients did not require colonoscopy [68]. It is important to highlight that both studies emphasize that colonoscopy should be performed in cases of complicated AD or the presence of alarming features.

A systematic review that deserves special attention is the one by Galetin et al., which compared national and international guidelines on the management of diverticular disease. In this review, where 11 guidelines were ultimately analyzed, it is noted that most clinical guidelines recommend follow-up colonoscopy, except those issued by the Netherlands Society of Surgeons (NSS) and the World Society of Emergency Surgery (WSES). These guidelines incorporated nuances regarding the indications for performing endoscopy. What stands out in this paper is that, upon closer examination, it is clear there is hardly any detailed discussion or justification for this recommendation in the guidelines, and the same articles are repeatedly cited to support the decision [69].

In addition to these key publications, a large number of studies of varying quality and levels of evidence have been published since 2010 with the aim of evaluating the utility of colonoscopy following an episode of AD. Although most of these studies are retrospective, they generally tend to conclude that systematic colonoscopy is unnecessary except in cases of persistent symptoms, the presence of abscesses or stenosis, or CT findings suggestive of a neoplastic process [70,71,72].

Three studies are particularly notable for their conclusions. The first, conducted by Horesh et al., concluded that not only was CRC rarely detected in these colonoscopies, but that performing colonoscopy was even more questionable in patients under 50 years of age [73]. The second study was a multicenter retrospective case–control study conducted in France, comparing patients with AD to individuals undergoing routine CRC screening. This study found no significant differences in the detection of adenomas or CRC between the two groups and, notably, reported a lower rate of advanced adenomas in the AD group. The study recommended endoscopy only for patients with suspicious CT findings, alarm symptoms, or other CRC risk factors [74]. Finally, the study by Schout et al. concluded that if colonoscopy had been limited solely to patients presenting with alarm symptoms, only 0.5% of CRC cases would have been missed [75]. Although no one desires such an outcome, from an epidemiological perspective, these figures seem more than reasonable, especially considering that appointment slots could be reallocated to prioritize endoscopies for patients with alarm signs, whether for CRC or other serious conditions.

Although some articles still advocate for maintaining colonoscopy after an episode of AD, as previously discussed, recent years have seen the continued emergence of studies questioning its necessity and emphasizing the need to carefully select patients for this procedure [47,76,77]. The study conducted by Mäntymäki et al., which followed a cohort of 270 patients for up to 18 years, deserves special mention. It found that patients with uncomplicated AD had a very low risk of CRC both in the short and long term, whereas patients with complicated AD, alarm symptoms, or persistent symptoms benefited from undergoing colonoscopy after the AD episode [78]

In addition to reaffirming the indications for colonoscopy in patients with complicated AD, persistent symptoms, or suspicious CT findings, several studies have begun to identify specific clinical scenarios to better select patients who would benefit from endoscopy. One study suggested a greater benefit among patients over 70 years of age with complicated AD [79]. Another study by Albshesh et al. concluded that if a patient had undergone colonoscopy within the five years prior to the AD episode, repeating the procedure provided no additional benefit; moreover, CRC was only detected in patients over 70 years old with complicated AD [80]. Lastly, a large cross-sectional study including nearly 92,000 post-diverticulitis follow-up colonoscopies and more than 4.5 million CRC screening colonoscopies found that the risk of CRC after an episode of AD was low, recommending colonoscopy only for patients with complications or those who had not previously undergone CRC screening [81].

All this growing evidence should prompt reflection on our daily clinical practice, encouraging a more individualized approach to the management of these patients to maximize their benefit while also improving resource allocation. Furthermore, it should drive further research in this field, as the current evidence is mainly based on retrospective studies, limiting the strength of clinical guideline recommendations.

## 5. Discussion

Like diverticulosis, AD is a growing condition. Population aging and increasing obesity rates are among the main drivers of this phenomenon. Paradoxically, a growing number of cases are now diagnosed in younger individuals under the age of 50, as reported by several studies [82,83]. Lifestyle changes, dietary habits, and tobacco consumption have been identified as potential drivers of these trends.

The rising incidence and prevalence of AD in the general population have important healthcare implications. If the traditional recommendation of routinely performing colonoscopy after each episode of AD were maintained, the resulting increase in endoscopic procedures would likely overwhelm endoscopy units. This would exacerbate scheduling delays and negatively affect the timely diagnosis and management of other high-priority gastrointestinal conditions.

Although some studies continue to advocate for routine colonoscopy following an episode of AD, a growing body of evidence has challenged this practice over the past two decades. This shift highlights the need for a more individualized approach, tailoring decisions based on patient-specific risk factors.

Numerous meta-analyses and systematic reviews published since 2010 have demonstrated that the prevalence of CRC following an episode of uncomplicated diverticulitis, particularly in the absence of alarm features, persistent symptoms, or CT findings suggestive of malignancy, is comparable to that observed in the general screening population. Conversely, complicated diverticulitis characterized by abscesses, strictures, or diverticular bleeding is associated with a markedly increased risk of relevant neoplastic findings, thus justifying colonoscopy evaluation in these cases.

**Figure 1:**
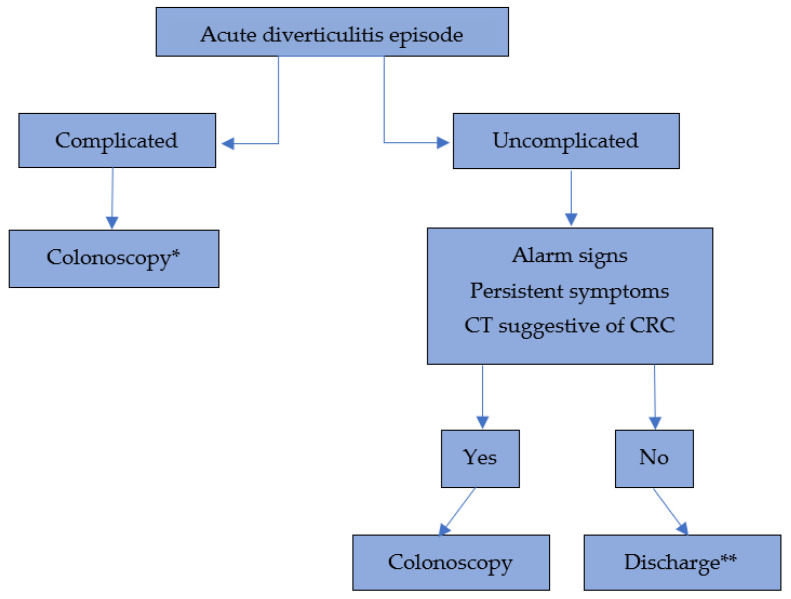
Proposed decision-making algorithm after an episode of acute diverticulitis. CT: Computed Tomography. CRC: Colorectal Cancer. * consider not performing colonoscopy if a high-quality colonoscopy was done within the last year or if there have been repeated episodes with normal colonoscopy results. ** consider colonoscopy if there is no prior colonoscopy within the last 5 years or if it is the first episode of diverticulitis. Evaluate risk/benefit and assess each case individually.

This evolving evidence base has been progressively reflected in national and international clinical guidelines. Earlier guidelines uniformly recommended post-AD colonoscopy for all patients. However, more recent versions have refined these indications, limiting it to higher-risk subgroups. Since 2020, the main guidelines have updated their recommendations, specifically addressing episodes of uncomplicated diverticulitis. ESC highlights the existing controversy and suggests that post-AD colonoscopy may not always be necessary [20]; WSES advises against routine colonoscopy following uncomplicated AD [84]; the American Society of Colon and Rectal Surgeons (ASCRS) guidelines on left-sided colonic diverticulitis state that even in cases of complicated diverticulitis, colonoscopy may be avoided if a recent, high-quality colonoscopy has been performed, implicitly suggesting a surveillance interval comparable to standard CRC screening (approximately five years) [85]; the American College of Physicians (ACP) also recommends avoiding systematic colonoscopy in cases of left-sided AD [86]. The American Gastroenterological Association (AGA) recommends colonoscopy only after the first episode of uncomplicated diverticulitis, considering the patient’s clinical history and whether a high-quality colonoscopy has been performed recently (ideally within the past year) [19]. The AGA guidelines also state that even in cases of complicated diverticulitis, the absence of alarm features and the existence of a recent high-quality colonoscopy may allow colonoscopy to be safely omitted. Figure 1 attempts to summarize the approach to be followed after an episode of acute diverticulitis based on the evidence gathered in this review.

Regarding the optimal timing for colonoscopy, current recommendations advise delaying the procedure for 6 to 8 weeks after complete clinical and radiological resolution of inflammation. In some cases, repeat imaging may be warranted to confirm resolution prior to endoscopy. This precaution is critical, as persistent inflammation may increase the risk of procedural complications [19]. Furthermore, colonoscopy itself may trigger recurrent diverticulitis in susceptible individuals, underscoring the importance of ensuring complete recovery before proceeding [87].

Despite these insights, most available evidence derives from retrospective studies, often characterized by significant heterogeneity in inclusion criteria, outcome definitions, and risk stratification. Prospective multicenter cohort studies and randomized controlled trials are urgently needed to validate risk thresholds for CRC detection in the context of AD.

In parallel, research into novel risk stratification tools, including biomarkers, gut microbiome profiles, and non-invasive imaging modalities, may enhance clinical decision-making. Promising data have emerged regarding the use of fecal immunochemical testing (FIT) in patients with uncomplicated diverticulitis, suggesting that a negative result may safely obviate the need for colonoscopy [88,89]. Furthermore, an innovative study by Ziegelmayer et al. employed a deep learning model to improve diagnostic accuracy for CRC detection following AD [90].

Future research in these areas holds the potential to advance personalized management strategies for patients with AD. Multidisciplinary collaboration among gastroenterologists, colorectal surgeons, and radiologists will be pivotal to driving further progress.

## 6. Conclusions

Current evidence consistently supports a paradigm shift in the management of patients following an episode of AD. In contrast to the historical practice of systematically recommending colonoscopy, a more rational and selective approach is now advocated, based on each patient’s individual risk profile.

Colonoscopy after an episode of AD should be selectively indicated and reserved for patients who present with complications identified on CT imaging, persistent alarm symptoms (such as abdominal pain, unexplained weight loss, altered bowel habits, hematochezia, or iron-deficiency anemia), or imaging findings suggestive of neoplasia. In cases of uncomplicated diverticulitis without alarm features, routine colonoscopy is not justified, as its systematic use may expose patients to unnecessary procedural risks and contribute to the inefficient allocation of healthcare resources.

Given the limitations in the quality of the studies supporting current recommendations, continued research is crucial to strengthen the evidence base and achieve broader consensus. Additionally, further investigation into non-invasive diagnostic strategies and the management of complicated diverticulitis represent promising areas for advancing individualized patient care.

Finally, it is imperative to promote the development of prospective studies aimed at validating current risk-based criteria and exploring novel risk stratification tools for this patient population.

## Figures and Tables

**Table 1:** Summary of treatment for patients with complicated acute diverticulitis based on the Hinchey classification. Percentages are estimates from various studies and may vary depending on the sources consulted. The antibiotics to be used should cover Gram-negative bacteria and anaerobes [42,43,44,45,46].

Hinchey	Description	Estimated Percentage	Treatment
Ia	Pericolic inflammation or phlegmon	45–55%	Antibiotics
Ib	Small pericolic abscess	5–15%	Antibiotics. Consider percutaneous drainage if >4–5 cm
II	Pelvic, intraabdominal, or retroperitoneal abscess	15–30%	Antibiotics. Consider percutaneous drainage if >4–5 cm
III	Generalized purulent peritonitis	5–15%	Emergency surgery (primary resection and anastomosis or Hartmann)
IV	Purulent fecal peritonitis	5–10%	Emergency surgery (Hartmann)

## Data Availability

Not applicable.
